# Racial/Ethnic Disparities in US Adolescents’ Dietary Quality and Its Modification by Weight-Related Factors and Physical Activity

**DOI:** 10.3390/ijerph16234803

**Published:** 2019-11-29

**Authors:** Furong Xu, Steven A. Cohen, Mary L. Greaney, Disa L. Hatfield, Geoffrey W. Greene

**Affiliations:** 1 Department of Kinesiology, University of Rhode Island, Independence Square II, Kingston, RI 02881, USA; doch@uri.edu; 2 Department of Health Studies, University of Rhode Island, Independence Square II, Kingston, RI 02881, USA; steven_cohen@uri.edu (S.A.C.); mgreaney@uri.edu (M.L.G.); 3 Department of Nutrition and Food Sciences, University of Rhode Island, Fogarty Hall, Kingston, RI 02881, USA; ggreene@uri.edu

**Keywords:** adolescents, racial/ethnic disparities, dietary quality, physical activity

## Abstract

There are well-known disparities in the prevalence of obesity across racial-ethnic groups, although the behavioral and psychological factors driving these disparities are less well understood. Therefore, the objectives of this study were: (1) to examine differences in dietary quality by race/ethnicity and weight-related variables [body mass index (BMI), weight loss attempt, and weight dissatisfaction] and physical activity (PA) using the Health Eating Index-2015 (HEI-2015); and (2) to investigate the interactions and independent associations of race/ethnicity, weight-related variables and PA on dietary quality. Data for adolescents aged 12–19 years (*n* = 3373) were abstracted from the 2007–2014 National Health and Nutrition and Examination Survey and analyzed using multiple PROC SURVEYREG, adjusting for demographics and accounting for complex sampling. Analyses determined that Hispanic males had better overall HEI-2015 scores than non-Hispanic whites (48.4 ± 0.5 vs. 45.7 ± 0.6, *p* = 0.003) or blacks (48.4 ± 0.5 vs. 45.5 ± 0.5, *p* < 0.001). Hispanic females also had better dietary quality than non-Hispanic whites (50.2 ± 0.4 vs. 47.5 ± 0.5, *p* < 0.001) and blacks (50.2 ± 0.4 vs. 47.1 ± 0.5, *p* < 0.001). Meeting the PA recommendation modified racial/ethnic differences in dietary quality for females (*p* = 0.011) and this was primarily driven by the associations among non-Hispanic white females (ΔR^2^ = 2.6%, *p* = 0.0004). The study identified racial/ethnic and gender differences among adolescents in factors that may promote obesity. Results may be useful for obesity prevention efforts designed to reduce health disparities in adolescents.

## 1. Introduction

Obesity among adolescence is a concern as 20.6% of US adolescents aged 12–19 have obesity [1]. Obesity during adolescence is associated with many physical and mental health conditions and may have long-term impacts on health status in adulthood [2]. Research has indicated that diet quality, consuming a healthy and balanced diet, may be an important strategy for obesity prevention due to its relationship with overweight or obesity in adolescents [3,4,5]. However, only limited research has examined dietary quality in adolescents [6,7,8], and, to our knowledge, only one study has explored racial/ethnic differences in dietary quality [8]. This study assessed diet used the Healthy Eating Index-2010 (HEI-2010) for children and adolescents aged 2–18 and found that Mexican Americans had better dietary quality than other racial/ethnic groups while non-Hispanic blacks had the poorest dietary quality [8]. Given racial/ethnic differences in obesity rates [1], it is important to understand differences in dietary quality by race/ethnicity using current dietary guidelines. The 2015–2020 guidelines focus on the totality of healthy eating patterns and for the first time include a recommendation for limiting added sugars and saturated fats (<10% of total daily caloric intake) [9]. The 2015–2020 guidelines also brought two additional changes to the HEI-2015, which is the latest version of dietary quality measure, regarding how energy from alcohol accounted for and how legumes allocated to different dietary components [9]. No studies, to the best of our knowledge, have examined racial/ethnic disparities in adolescents’ dietary quality as assessed using the HEI-2015 which aligns with 2015–2020 dietary guidelines and MyPlate dietary recommendations.

Research has identified a negative relationship between body mass index (BMI) and eating a prudent dietary pattern, which is defined by high consumption of vegetables, legumes, nuts and seeds, fruits, and whole grains [10]. Studies have identified a correlation between weight loss attempts and eating less fatty foods or fewer calories and protein among males only [11,12]. Studies have also identified an inverse relationship between weight dissatisfaction and the Western pattern diet consumption [13]; and associations between higher levels of physical activity and lower intake of saturated fat and higher intake of fruits and vegetables, and dairy products [14,15]. Most of these studies, however, have assessed dietary quality using measures that are less comprehensive than the HEI-2015 [10,13], or assessed only selected aspects of diet [11,12,14,15], and/or did not control for sociodemographic characteristics [10,14,15]. No identified studies have examined differences in dietary quality as assessed by the HEI-2015 by weight-related factors and physical activity controlling for sociodemographic characteristics.

Research is needed to understand dietary quality differences by race/ethnicity, and weight-related variables and physical activity among US adolescents using the HEI-2015. In addition, the influence of sociodemographic characteristics, and weight-related factors and physical activity on dietary quality in adolescents is poorly understood. This research may lead to more effective interventions for obesity prevention efforts designed to reduce racial/ethnic health disparities in adolescents. Accordingly, the purpose of the present study was to systematically explore racial/ethnic and weight-related and physical activity differences in dietary quality and its interactions and independent associations on dietary quality through a progressive set of hypotheses and exploratory analyses using a representative sample of US adolescents and the HEI-2015. Due to the previously identified sex effects [4,8], all analyses were stratified by sex.

## 2. Methods

### 2.1. Study Design and Participants

The present study is a cross-sectional analysis of data from the 2007–2014 National Health and Nutrition and Examination Survey (NHANES) and the 2007–2014 US Department of Agriculture’s Food Patterns Equivalents database [16,17]. Of 40,617 respondents, 5282 were aged 12–19, of whom 3927 had completed data on weight satisfaction, physical activity, and two 24-h dietary recalls [16,17]. Of these respondents, 117 (3.0%) were underweight and were excluded from the study due to weight-related psychological or physical pathology concerns [18]. In addition, 437 (7.7%) respondents whose race/ethnicity was classified as “other” were excluded due to the limited sample size, heterogeneity of this category, and to be consistent with other research [16,19]. The final analytical sample was 3373 adolescents aged 12–19. This study has been reviewed and approved by the University of Rhode Island’s Institutional Review Board (IRB) under exempt review category according to federal regulation 45 CFR 46 (IRB#1500884-1).

### 2.2. Diet Quality

Dietary quality was calculated using the National Cancer Institute’s simple HEI-2015 scoring algorithm and data collected from two 24-h dietary recalls [16,20]. Thirteen dietary component scores were calculated (fatty acids, total vegetables, greens and beans, total fruit, whole fruit, whole grains, dairy, total protein foods, seafood and plant proteins, refined grains, sodium, saturated fats, and added sugars) [20]. Each component was scored on density out of 1000 calories independent of quantity except fatty acids which used the ratio of unsaturated to saturated fatty acids [20]. Then, the 13 component scores were summed to create a total dietary quality score (range: 0–100).

### 2.3. Physical Activity

Physical activity was measured across the three domains of work, travel, and recreational using the Global Physical Activity Questionnaire (GPAQ) [16,21]. The metabolic equivalent of task (MET) minutes in each domain were summed to create weekly MET minutes of moderate to vigorous physical activity [16]. Respondents aged 12–17 were classified as meeting the physical activity recommendation if they participated in 1680 MET-minutes of activity per week while respondents aged 18–19 were classified as meeting the recommendation if they reported 600 MET-minutes or more per week [22].

### 2.4. Weight Loss Attempts

Respondents aged 12–15 years reported how often they tried to lose weight in the past year (never, sometimes, a lot) while respondents aged 16–19 years reported if they tried to lose weight in the past year (yes, no) [16]. Individuals who answered “sometimes”, “a lot” (aged 12–15 years), or “yes” (aged 16–19 years) were classified as attempting to lose weight [23].

### 2.5. Weight Satisfaction

Weight satisfaction was assessed by one item that asked respondents “which of the following are you trying to do about your weight?” with four options: (1) lose weight; (2) gain weight; (3) stay the same weight; and (4) not trying to do anything about your weight. Respondents who reported wanting to lose weight or gain weight were classified as being weight dissatisfied. Respondents were classified as being weight satisfied if they reported that they wanted to stay the same weight or were not trying to do anything about their weight [4,16].

### 2.6. Body Mass Index (BMI) and Weight Status

BMI was retrieved directly from the 2011–2014 NHANES dataset and had been calculated using measured height (meters) and weight (kilograms) [16]. Whereas for 2007–2010 NHANES dataset, the SAS program for 2000 Centers for Disease Control and Prevention growth charts was used to calculate sex and age specific BMI using measured height and weight [16].

### 2.7. Demographic Characteristics

Respondents reported their race/ethnicity (Mexican American, other Hispanic, non-Hispanic white, non-Hispanic black, Other) [16]. This information was used to create three categories: (1) non-Hispanic white; (2) non-Hispanic black; and (3) Hispanic (Mexican American and other Hispanic) [1,16].

Additional examined demographic characteristics included age (in years), sex, parent education (high school or less, college or above), and the ratio of family income to poverty (PIR) [16]. PIR was calculated by using family income and family size and then categorized into two household income categories: (1) PIR ≥ 1: at or above the poverty level; and (2) PIR < 1: below the poverty level [24].

### 2.8. Data Analysis

All analyses were conducted by using the Mobile Examination Center (MEC) exam 2-year weights as recommend by the National Center for Health Statistics when using NHANES data due to unequal sample selection probability [25]. Analyses were conducted using SAS version 9.4 (SAS Institute Inc., Cary, NC, USA) and significance was set at *p* < 0.05. Descriptive results were obtained for the sample and are presented as weighted means ± standard error for continuous variables and frequencies and weighted proportions for categorical variables. The following specific analyses were conducted.

#### 2.8.1. Differences in Dietary Quality by Demographic Characteristics, Weight-Related Variables, and Physical Activity

*p*-values for continuous variables were obtained by performing PROC SURVEYREG (linear regression) while *p*-values for categorical variables were obtained by performing PROC SURVEYLOGISTC (logistic regression), with STRATA, CLUSTER, and WEIGHT statement in these two types of models, to perform the adjusted analyses (adjusted for age, sex, parental education level and PIR) [26,27]. The only exceptions were for sex, age, parental education level, and PIR comparisons between racial/ethnic groups, which were performed without adjusted analysis as these are not the main predictor variables of interest in the overarching research question addressed in this study. Bonferroni correction was applied to all multiple comparisons between racial/ethnic groups or weight status. In addition, to correct familywise error rates and to be consistent with another research, the Bonferroni critical value for the 13 components was set at *p* < 0.003846, which is 0.05/13 (number of components) [28].

#### 2.8.2. Effect Modification of Weight-Related Variables and Physical Activity on Racial/Ethnic Differences in Dietary Quality

In the initial exploration of data, the four primary variables (BMI, weight loss attempt, weight dissatisfaction, and physical activity), race/ethnicity, and the interaction terms for the four primary variables and race/ethnicity were analyzed as indicator variables fitting four separated models for each outcome with the use of PROC SURVEYREG. Variables included in this initial exploratory analysis were considered in the multivariate model, with age, parental education level, and PIR being included as covariates [26,27] in all the models. Since PROC SURVEYREG does not have the capacity of automated model selection, variables selection was done manually by removing the non-significant variables step by step until there were only variables with *p* < 0.05 (physical activity). Race/ethnicity was included in the final model as it is the primary independent variable.

#### 2.8.3. Percent Variability of Total HEI-2015 Scores Explained by Predictor Variables

We used sequence models to look at R-squared, the percentage of variability in the outcome explained by the predictor variable(s), by performing multiple PROC REG with the WEIGHT statement to account for sample weights to identify the most important predictor variables in regression models. For independent effects of race/ethnicity, weight-related variables and physical activity on total HEI-2015 scores, the predictor variables were entered sequentially as follows (1) age, sex, parental education level, and PIR; (2) weight-related variables and physical activity; and (3) race/ethnicity. To determine the independent effects of weight-related variables and physical activity on the total HEI-2015 scores stratified by race/ethnicity, predictor variables were entered sequentially as follows: (1) age, sex, parental education level, and PIR; (2) BMI; (3) weight loss attempt; (4) weight dissatisfaction; and (5) physical activity. Variables were entered into the model in a sequence that was aligned with the priority of the present study and prior research [8,10,11,12,13,14,15,26,27]. The change in R-squared (ΔR^2^) calculated the increase in R-squared that each variable produces when added to a model containing all of the other variables. Root MSE were used to test the model goodness-of-fit, and F value and Pr > F (probability of the F statistic) were assessed to determine whether the group of independent variables could be used to reliably predict the dependent variable.

## 3. Results

Of the 3373 respondents, approximately half (49.5%) were females, 37.1% were racial/ethnic minorities, 41.4% of the sample had parents who had a high school diploma or less, and 21.8% had a PIR below poverty level. In total, 17% of respondents were classified as being overweight, 20.9% had obesity, 51.3% were weight dissatisfied, 41.6% reported trying to lose weight in the last year, and 60% met the physical activity recommendation. Non-Hispanic blacks and Hispanics were more likely to live in a household with an income below the poverty level than non-Hispanic whites (*p* < 0.001) and more likely to have parents with less than a high school education than non-Hispanic whites (*p* < 0.001). See Table 1 for sample characteristics stratified by race/ethnicity.

Controlling for age, sex, income, and parental education, a greater proportion of non-Hispanic whites had a lower BMI, were classified as normal weight, and met PA recommendations than non-Hispanic blacks and Hispanics. A smaller proportion of non-Hispanic whites were classified as being weight dissatisfied, and reported a weight loss attempt in the previous year than non-Hispanic blacks and Hispanics (see Table 1). Additionally, there were differences in most dietary component scores and weight-related variables and physical activity by sex (see Table S1).

### 3.1. Dietary Quality Differences by Race/Ethnicity

Dietary quality by race/ethnicity is presented in Table 2. Overall, the weighted mean dietary quality scores, controlling for sociodemographic characteristics for all racial/ethnic groups, were low (total HEI-2015 score < 50 out of 100). Hispanic males (48.4 ± 0.5) and females (50.2 ± 0.4) had a higher total diet quality score than non-Hispanic whites [45.7 ± 0.6, *p* = 0.005(males); 47.5 ± 0.5, *p* < 0.001 (females)] and non-Hispanic blacks [45.5 ± 0.5, *p* < 0.001 (males); 47.1 ± 0.5, *p* < 0.001 (females)].

Examining the 13 dietary components, after controlling for sociodemographic variables and correcting for multiple comparisons, Hispanic males had the highest component scores for greens and beans (*p* < 0.001), seafood and plant proteins (*p* < 0.001), and added sugars (*p* < 0.001), as did Hispanic females. There was also difference in intake of refined grains by sex; non-Hispanic white and black males had higher scores than Hispanic males (*p* < 0.001) while non-Hispanic black females had higher scores than Hispanic females (*p* = 0.002).

### 3.2. Dietary Quality Differences by Weight-Related Variables and Physical Activity

There was no difference in total HEI-2015 scores by the examined weight-related variables by sex (see Table 3). There also were no significant differences in total HEI-2015 scores in the effect of meeting physical activity guidelines for males. However, females who met the physical activity recommendation had a higher dietary quality score (49.3 ± 0.6) than those not meeting the recommendation (46.8 ± 0.5; *p* = 0.004).

The multivariable analysis of the 13 components of dietary quality among males, after controlling for sociodemographic variables, found that male respondents with obesity scored higher in refined grains (5.4 ± 0.2) than those who were normal weight (4.7 ± 0.1, *p* = 0.003). Additionally, respondents who were weight dissatisfied had higher intake of total protein foods (*p* = 0.016) as well as seafood and plant proteins (*p* = 0.048) than those who were weight satisfied. Respondents who met physical activity recommendations had higher intake of total fruits (*p* = 0.004) than those not meeting the recommendation.

There were four dietary components scores that differed by the examined weight-related variables and physical activity in females. Females who were satisfied with their weight scored higher (5.2 ± 0.1) than those who were dissatisfied with their weight (4.6 ± 0.2, *p* < 0.001) for refined grains. However, respondents who were dissatisfied with their weight scored higher for added sugar than those who were satisfied with their weight (5.7 ± 0.1 vs. 5.1 ± 0.1, *p* = 0.002). Moreover, respondents who met physical activity recommendation scored higher than those who did not meet the recommendation for total fruits (2.5 ± 0.1 vs. 2.1 ± 0.1, *p* = 0.004) and whole fruits (2.4 ± 0.1 vs., 2.0 ± 0.1, *p* = 0.012).

### 3.3. Effect Modification of Weight-Related Variables and Physical Activity on Total HEI-2015 Score Differences by Race/Ethnicity

Meeting the physical activity recommendation in females modified dietary quality differences by race/ethnicity (*p* = 0.011). There were no other significant interactions between the weight-related variables, physical activity, and race/ethnicity on total dietary quality score (see Table 4).

Further analysis determined that non-Hispanic white females who did not meet the physical activity recommendation had lower total dietary quality by 6.43 points than Hispanic females, whereas non-Hispanic white females who met the recommendation had lower total dietary quality by 1.34 points (see Table 5).

### 3.4. Percent Variability of Total HEI-2015 Scores Explained by Predictor Variables

Table 6 presents the associations between race/ethnicity and weight-related variables and physical activity on total HEI-2015 scores. Among males, race/ethnicity was the only significant independent predictor of dietary quality (ΔR^2^ = 0.8%, *p* = 0.0004) and the overall model only explained 1.7% of the variance. Among females, in addition to race/ethnicity (ΔR^2^ = 1.3%, *p* < 0.0001), other sociodemographic characteristics (ΔR^2^ = 0.9%, *p* = 0.0031), weight-related variables, and physical activity (ΔR^2^ = 1.4%, *p* = 0.0014) also were significant predictors and the overall model explained 3.6% of the variance in dietary quality.

To better understand the associations of sociodemographic, weight-related variables, and physical activity on total HEI-2015 scores, we conducted a stratified regression analyses (see Table 7), which determined that only a small proportion of the variance of total HEI-2015 score was explained by the models. For males, there was only one significant predictor, weight dissatisfaction among non-Hispanic whites (ΔR^2^ = 0.8%, *p* = 0.0456). For females, sociodemographic variables (ΔR^2^ = 3.6%, *p* = 0.0008) and meeting physical activity recommendations (ΔR^2^ = 2.6%, *p* = 0.0004) were significant predictors of HEI-2015 score in non-Hispanic white females but no variables were significant for non-Hispanic black or Hispanic females.

## 4. Discussion

To the authors’ knowledge, this is the first study to systematically assess diet quality using the HEI-2015 in a representative sample of US adolescents and to examine the effect of race/ethnicity, weight-related factors and physical activity. The study determined that Hispanic adolescents, regardless of sex, had a higher total diet quality score than non-Hispanic whites or blacks, which reinforces findings from previous research [8]. Meeting the physical activity recommendations was associated with improved dietary quality in females, and this was primarily driven by the associations among non-Hispanic white females. The overall variance in total HEI-2015 scores explained by study variables was in the low to moderate range [29], but race/ethnicity was a significant independent predictor of dietary quality for males and females, whereas weight-related factors and physical activity were independent predictors of dietary quality for females only. This study extended previous research and findings can be used to inform interventions for adolescents. Specifically, the present study validated findings from previous research and extended these findings by using the HEI-2015, and focused on adolescents who have been found in other studies to have a lower dietary quality than children [8,30]. The present study also stratified analyses by sex due to consistent sex differences found in other studies [4,8]. Importantly, the present study controlled for sociodemographic characteristics and, through regression analyses determined the unique contribution of race/ethnicity and weight-related factors and physical activity to total HEI-2015 scores.

The present study found that Hispanics had a higher HEI-2015 score than non-Hispanic whites and blacks. These results are consistent with Gu and Tucker’s analyses of 1999–2012 NHANES data using the HEI-2010 [8] that determined that Mexican Americans children had higher HEI-2010 scores than non-Hispanic white and black children [8]. The present study also found that Hispanic males and/or females had dietary patterns that are indicative of a greater consumption of fruits, greens and beans, seafood and plant proteins and less added sugars, but less healthful grain choices than non-Hispanic whites and blacks. It should be noted that the HEI-2015, unlike previous versions, included legumes in total vegetables, greens and beans, total protein foods, and seafood and plant proteins as well as a new category for added sugars [9,20]. Nevertheless, study results are broadly consistent with previous research [31,32,33]. It is possible that the pattern of higher dietary quality in Hispanics is related to foods traditionally consumed (e.g., legumes, fruits, and vegetables) by Hispanics [33,34]. It also is important to note that most respondents had total HEI-2015 scores and the majority of dietary components scores less than 50% of the recommendations, indicating a pattern of poor adherence to the 2015–2020 Dietary Guidelines for Americans [35]. The scores for greens and beans and whole grains were particularly low with around 20% of maximum scores. These findings indicate a need for interventions in this age group to develop healthier eating habits that may persist into adulthood.

Furthermore, the present study found HEI-2015 scores differed by physical activity in females, whereas intake of some dietary components (e.g., total vegetables, total protein foods, and refined grains) differed by weight-related variables and physical activity in both males and females. Previous research has found dietary quality among adolescents is associated with weight-related factors (e.g., weight dissatisfaction and weight loss attempts) and physical activity. However, due to the use of different dietary quality measures, it is challenging to make cross-study comparisons [10,11,12,13,14,15]. This study extended existing research by exploring whether weight-related variables and physical activity modified the dietary quality differences between racial/ethnic groups and its independent associations on dietary quality. Analyses determined that meeting physical activity recommendation modified dietary quality difference between non-Hispanic white females and Hispanic females. Analyses also determined that meeting physical activity recommendation was associated with dietary quality among non-Hispanic white females. This finding suggests that non-Hispanic white females who were physically active might be more aware of the need for physical activity and healthy diet than their underactive counterparts. No other weight-related variables were found to moderate dietary quality differences between racial/ethnic groups, although weight dissatisfaction had a direct association with non-Hispanic white males’ dietary quality. A possible explanation for this finding is that non-Hispanic white males who are weight dissatisfied pay greater attention to their dietary quality, but such differences did not moderate dietary quality differences between non-Hispanic white males and other racial/ethnic groups. Further research is needed to investigate the potential influence of examined weight-related factors on racial/ethnic disparities in dietary quality in adolescents. Nevertheless, study findings indicated race/ethnicity is associated with dietary quality for both males and females, whereas meeting physical activity recommendation had an interaction and independent association with dietary quality only among females.

A practical implication of the present study is that it provides information on factors that might be related to dietary choices, which can be used to inform intervention to promote a healthful diet and help prevent obesity in adolescence thus reducing health disparities related to race/ethnicity [3,4,5]. Health practitioners can work with adolescents, parents, and policy makers to advocate for more healthful food choices and to increase awareness of healthful food options. Results of the present study also indicate that intervention efforts need to address racial/ethnic differences on dietary choices, suggesting a need for specific message tailoring. For example, Hispanic adolescents could be reinforced for their more healthful dietary patterns but encourage to shift to towards greater consumption of whole grains. The present study also raises a need for research examining the influence of weight-related factors and physical activity on dietary quality between racial/ethnic groups. A longitudinal design could examine whether positive changes in weight-related factors and physical activity in adolescents could improve dietary quality among racial/ethnic groups over time.

Study findings should be considered within the context of its strengths and limitations. A strength of this study is the use of nationally representative data on adolescents. The study adds to the existing literature on racial/ethnic disparities in dietary quality by demonstrating moderation of weight-related variables and physical activity. Another strength is utilizing the HEI-2015 to measure diet quality, which was aligned with the 2015–2020 Dietary Guidelines for Americans [9]. This study also has several important limitations. As a cross-sectional study, it is not possible to determine causal relationships [36]. Dietary intake was assessed through recall methodology, which may underestimate intake and there were limitations in assessment of weight-related variables and physical activity. Weight dissatisfaction and weight loss attempt in the previous year were assessed by validated single items, which are less reliable than scales [37]. Physical activity was collected via self-report; however, it was assessed by a widely used and validated instrument [21]. Finally, to clarify distinctions between racial/ethnic groups, the current study excluded the “Other” race category.

## 5. Conclusions

Hispanic adolescents had higher overall diet quality and higher quality intake for most dietary components compared to non-Hispanic whites and blacks but overall adolescents had poor dietary quality. Race/ethnicity was a significant predictor of total dietary quality after controlling for sociodemographic characteristics, weight-related variables, and physical activity. Meeting physical activity recommendations had a direct association with dietary quality as well as an interaction with racial/ethnic differences in dietary quality in females mainly driven by the associations among non-Hispanic females. Weight dissatisfaction was associated with dietary quality among non-Hispanic white males, although race/ethnicity did not moderate these associations. These results indicate that tailoring messages for healthful eating and physical activity may be an important strategy for improving the health of adolescents.

## Figures and Tables

**Table 1:** Sample characteristics stratified by race/ethnicity (*n* = 3373).

	Total	Non-Hispanic White	Non-Hispanic Black	Hispanic	*p*-Value
	*n* = 3373	*n* = 1082 (62.9%)	*n* = 968 (15.4%)	*n* = 1323 (21.7%)	
**Not adjusted analysis ^&,@^**					
Age (yrs)	15.4 ± 0.1	15.4 ± 0.1	15.5 ± 0.1	15.3 ± 0.1	0.400
Gender, *n* (weighted %)					
Male	1711 (50.5)	576 (50.9)	483 (49.1)	652 (50.2)	0.676
Female	1662 (49.5)	506 (49.1)	485 (50.9)	671 (49.8)	0.676
Parent education level, *n* (weighted %)					
high school or less	1706 (41.4)	368 (29.9) ^a,b^	436 (46.4) ^c^	902 (70.6)	<0.001 *
college or above	1554 (58.6)	672 (70.1) ^a,b^	503 (53.6) ^c^	379 (29.4)	<0.001 *
PIR, n (weighted %)					
<1.0	1010 (21.8)	204 (12.7) ^a,b^	319 (35.1)	487 (40.1)	<0.001 *
≥1.0	2107 (78.2)	828 (87.3) ^a,b^	576 (64.9)	703 (59.9)	<0.001 *
**Adjusted analysis ^#,@^**					
Body mass index (kg/m^2^)	24.2 ± 0.2	23.7 ± 0.2 ^a^	25.3 ± 0.3	24.7 ± 0.2	0.006 *
Actual weight status, *n* (weighted %)					
Normal weight	1975 (62.1)	700 (65.8) ^a^	550 (56.7)	725 (55.3)	0.029 *
Overweight	613 (17.0)	166 (15.7)	174 (18.2)	273 (19.9)	0.139
Obese	785 (20.9)	216 (18.5)	244 (25.2)	325 (24.8)	0.233
Weight loss attempt (weighted %)	1504 (41.6)	421 (38.1) ^b^	409 (42.2) ^c^	674 (51.6)	<0.001 *
Weight dissatisfied, *n* (weighted %)	1872 (51.3)	516 (46.6) ^a,b^	560 (58.0)	796 (60.4)	<0.001 *
Met PA recommendation, *n* (weighted %)	1858 (60.0)	670 (64.5) ^a,b^	496 (51.9)	692 (52.7)	<0.001 *

Note: Data are present as weighted mean ± standard error for continuous variables and frequencies and weighted proportions for categorical variables; PA, physical activity; PIR, the ratio of family income to poverty. ^a.^ Non-Hispanic whites different from Non-Hispanic blacks with *p* < 0.05; ^b.^ Non-Hispanic whites different from Hispanics with *p* < 0.05; ^c.^ Non-Hispanic blacks different from Hispanics with *p* < 0.05. ^#^ *p*-value for continuous variables was obtained by performing PROC SURVEYREG, and *p*-value for categorical variable was obtained by performing PROC SURVEYLOGISTC, adjusted for sex, age, parental education level, and family income; ^&^ the analyses were done same as above adjusted analyses (^#^) except no adjustment for sex, age, parental education level, and family income; ^@^ Bonferroni correction was applied to all multiple comparisons between racial/ethnic groups with *p* < 0.05; * *p* < 0.05.

**Table 2:** Diet quality differences stratified by race/ethnicity (*n* = 3373).

	Total	Non-Hispanic White	Non-Hispanic Black	Hispanic	*p*-Value
**Male ^@^ (*n* = 1711)**	***n* = 1711**	***n* = 576 (63.5%)**	***n* = 483 (15.0%)**	***n* = 652 (21.6%)**	
HEI-2015 (100)	46.3 ± 0.4	45.7 ± 0.6 ^b^	45.5 ± 0.5 ^c^	48.4 ± 0.5	<0.001 *
Adequacy					
Total Fruits (5)	2.1 ± 0.1	1.9 ± 0.1 ^b^	2.2 ± 0.1	2.4 ± 0.1	<0.001 *
Whole Fruits (5)	1.9 ± 0.1	1.9 ± 0.1	1.8 ± 0.1	2.2 ± 0.1	0.012
Total Vegetables (5)	2.2 ± 0.0	2.2 ± 0.1	2.2 ± 0.1	2.4 ± 0.0	0.008
Greens and Beans (5)	1.0 ± 0.1	0.8 ± 0.1 ^b^	0.9 ± 0.1 ^c^	1.7 ± 0.1	<0.001 *
Whole Grains (10)	2.3 ± 0.1	2.5 ± 0.1	1.9 ± 0.1	2.0 ± 0.1	0.048
Dairy (10)	7.0 ± 0.1	7.3 ± 0.1 ^a^	5.8 ± 0.2 ^c^	7.1 ± 0.1	<0.001 *
Total protein foods (5)	4.2 ± 0.0	4.2 ± 0.0	4.2 ± 0.1	4.3 ± 0.1	0.404
Seafood and Plant Proteins (5)	1.8 ± 0.1	1.7 ± 0.1 ^b^	1.6 ± 0.1 ^c^	2.3 ± 0.1	<0.001 *
Fatty Acids (10)	3.8 ± 0.1	3.6 ± 0.1 ^a^	4.6 ± 0.2	4.0 ± 0.1	0.002 *
Moderation					
Refined Grains (10)	4.8 ± 0.1	5.2 ± 0.2 ^b^	4.8 ± 0.1 ^c^	3.9 ± 0.1	<0.001 *
Sodium (10)	4.1 ± 0.1	4.0 ± 0.1	4.4 ± 0.1	4.1 ± 0.2	0.049
Added Sugars (10)	5.3 ± 0.1	5.0 ± 0.2 ^b^	5.2 ± 0.2 ^c^	6.1 ± 0.1	<0.001 *
Saturated Fats (10)	5.7 ± 0.1	5.6 ± 0.1	5.8 ± 0.2	5.7 ± 0.1	0.927
**Female ^@^ (*n* = 1662)**	***n* = 1662**	***n* = 506 (62.4%)**	***n* = 485 (15.8%)**	***n* = 671 (21.8%)**	
HEI-2015 (100)	48.0 ± 0.3	47.5 ± 0.5 ^b^	47.1 ± 0.5 ^c^	50.2 ± 0.4	<0.001 *
Adequacy					
Total Fruits (5)	2.3 ± 0.1	2.1 ± 0.1 ^b^	2.3 ± 0.1 ^c^	2.7 ± 0.1	<0.001 *
Whole Fruits (5)	2.2 ± 0.1	2.2 ± 0.1	1.7 ± 0.1 ^c^	2.4 ± 0.1	<0.001 *
Total Vegetables (5)	2.6 ± 0.0	2.6 ± 0.1	2.5 ± 0.1	2.7 ± 0.1	0.029
Greens and Beans (5)	1.3 ± 0.1	1.1 ± 0.1 ^b^	1.2 ± 0.1 ^c^	1.6 ± 0.1	<0.001 *
Whole Grains (10)	2.4 ± 0.1	2.5 ± 0.1	2.0 ± 0.1	2.3 ± 0.1	0.036
Dairy (10)	6.6 ± 0.1	7.0 ± 0.2 ^a^	5.4 ± 0.1 ^c^	6.4 ± 0.1	<0.001 *
Total protein foods (5)	4.0 ± 0.0	3.9 ± 0.1 ^a,b^	4.2 ± 0.0	4.2 ± 0.0	0.002 *
Seafood and Plant Proteins (5)	2.0 ± 0.1	1.9 ± 0.1 ^b^	1.8 ± 0.1 ^c^	2.3 ± 0.1	<0.001 *
Fatty Acids (10)	4.4 ± 0.1	4.2 ± 0.2 ^a^	5.3 ± 0.1 ^c^	4.5 ± 0.2	<0.001 *
Moderation					
Refined Grains (10)	4.9 ± 0.1	5.0 ± 0.2	5.3 ± 0.2 ^c^	4.4 ± 0.1	0.003 *
Sodium (10)	4.1 ± 0.1	4.1 ± 0.2	4.1 ± 0.1	4.3 ± 0.1	0.509
Added Sugars (10)	5.4 ± 0.1	5.2 ± 0.2 ^b^	5.3 ± 0.2 ^c^	6.0 ± 0.1	<0.001 *
Saturated Fats (10)	5.9 ± 0.1	5.8 ± 0.2	6.0 ± 0.2	6.1 ± 0.1	0.346

Note: *p*-value for continuous variables was obtained by performing PROC SURVEYREG, and *p*-value for categorical variable was obtained by performing PROC SURVEYLOGISTC to perform the adjusted analyses (adjusted for age, sex, parental education level, and the ratio of family income to poverty); HEI, the Healthy Eating Index; ^a.^ Non-Hispanic whites different from Non-Hispanic blacks with *p* < 0.003846; ^b.^ Non-Hispanic whites different from Hispanics with *p* < 0.003846; ^c.^ Non-Hispanic blacks different from Hispanics with *p* < 0.003846; ^@^ Bonferroni correction was applied to multiple comparisons between racial/ethnic groups with *p* < 0.003846 to be significant; * *p* < 0.003846.

**Table 3:** Diet quality differences stratified by weight-related variables and physical activity (*n* = 3373).

	Weight Status				Weight Loss Attempt			Weight Satisfaction			Met PA Recommendation		
	Normal	Overweight	Obese	*p*-Value ^#^	Yes	No	*p*-value	Yes	No	*p*-Value	Yes	No	*p*-Value
**Male ^&^ (*n* = 1711)**	***n* = 1021 (61.1%)**	***n* = 290 (17.0%)**	***n* = 400 (21.8%)**		***n* = 1063 (64.4%)**	***n* = 647 (35.6%)**		***n* = 785 (49.3%)**	***n* = 926 (50.7%)**		***n* = 599 (31.1%)**	***n* = 1112 (68.9%)**	
HEI-2015 (100)	46.3 ± 0.5	45.4 ± 0.8	46.8 ± 0.7	0.494	46.6 ± 0.6	46.1 ± 0.5	0.578	45.8 ± 0.4	46.7 ± 0.6	0.261	46.1 ± 0.5	46.6 ± 0.7	0.656
Adequacy													
Total Fruits (5)	2.1 ± 0.1	2.1 ± 0.1	1.9 ± 0.1	0.243	2.1 ± 0.1	2.0 ± 0.1	0.635	2.0 ± 0.1	2.1 ± 0.1	0.623	2.1 ± 0.1	1.9 ± 0.1	0.004 *
Whole Fruits (5)	2.0 ± 0.1	1.9 ± 0.2	1.8 ± 0.1	0.278	2.1 ± 0.1	1.9 ± 0.1	0.462	2.0 ± 0.1	1.9 ± 0.1	0.663	1.9 ± 0.1	1.9 ± 0.1	0.446
Total Vegetables (5)	2.2 ± 0.1	2.2 ± 0.1	2.4 ± 0.1	0.165	2.1 ± 0.1	2.3 ± 0.1	0.033 *	2.2 ± 0.1	2.3 ± 0.1	0.149	2.2 ± 0.1	2.3 ± 0.1	0.255
Greens and Beans (5)	1.1 ± 0.1	1.0 ± 0.1	1.0 ± 0.1	0.159	1.0 ± 0.1	1.0 ± 0.1	0.068	1.0 ± 0.1	1.1 ± 0.1	0.089	1.0 ± 0.1	1.1 ± 0.1	0.661
Whole Grains (10)	2.3 ± 0.1	2.0 ± 0.2	2.4 ± 0.2	0.254	2.4 ± 0.2	2.3 ± 0.1	0.995	2.2 ± 0.1	2.4 ± 0.1	0.487	2.3 ± 0.1	2.4 ± 0.2	0.634
Dairy (10)	7.0 ± 0.1	7.1 ± 0.2	6.9 ± 0.2	0.852	7.2 ± 0.1	6.9 ± 0.1	0.428	6.9 ± 0.1	7.1 ± 0.1	0.772	7.1 ± 0.1	6.9 ± 0.2	0.313
Total protein foods (5)	4.2 ± 0.0	4.2 ± 0.1	4.3 ± 0.1	0.069	4.2 ± 0.1	4.2 ± 0.0	0.662	4.2 ± 0.1	4.3 ± 0.0	0.016 *	4.2 ± 0.0	4.3 ± 0.1	0.12
Seafood and Plant Proteins (5)	1.9 ± 0.1	1.6 ± 0.1	1.7 ± 0.1	0.07	1.9 ± 0.1	1.8 ± 0.1	0.754	1.7 ± 0.1	1.9 ± 0.1	0.048 *	1.8 ± 0.1	1.8 ± 0.1	0.873
Fatty Acids (10)	3.7 ± 0.1	3.7 ± 0.2	4.2 ± 0.2	0.162	3.9 ± 0.2	3.8 ± 0.1	0.453	3.9 ± 0.1	3.7 ± 0.1	0.573	3.7 ± 0.1	4.2 ± 0.2	0.044 *
Moderation													
Refined Grains (10)	4.7 ± 0.1	4.6 ± 0.3	5.4 ± 0.2^a^	0.002 ^$^	4.8 ± 0.2	4.9 ± 0.1	0.763	4.8 ± 0.2	4.9 ± 0.2	0.689	4.9 ± 0.1	4.8 ± 0.2	0.57
Sodium (10)	4.1 ± 0.1	4.2 ± 0.2	3.9 ± 0.2	0.682	4.1 ± 0.1	4.0 ± 0.1	0.809	4.1 ± 0.1	4.0 ± 0.2	0.463	4.1 ± 0.1	4.0 ± 0.2	0.484
Added Sugars (10)	5.4 ± 0.1	5.2 ± 0.3	5.2 ± 0.2	0.738	5.4 ± 0.2	5.2 ± 0.1	0.524	5.3 ± 0.1	5.3 ± 0.1	0.478	5.3 ± 0.1	5.3 ± 0.1	0.998
Saturated Fats (10)	5.6 ± 0.1	5.6 ± 0.2	5.8 ± 0.2	0.778	5.7 ± 0.2	5.7 ± 0.1	0.895	5.6 ± 0.1	5.7 ± 0.1	0.471	5.6 ± 0.1	5.8 ± 0.1	0.491
**Female ^&^ (*n* = 1662)**	***n* = 954 (63.1%)**	***n* = 323 (17.0%)**	***n* = 385 (20.0%)**		***n* = 805 (52.2%)**	***n* = 857 (47.8%)**		***n* = 716 (48.0%)**	***n* = 946 (52.0%)**		***n* = 916 (49.1%)**	***n* = 746 (50.9%)**	
HEI-2015 (100)	48.1 ± 0.5	47.6 ± 0.8	48.3 ± 1.0	0.908	48.3 ± 0.5	47.8 ± 0.5	0.578	47.8 ± 0.5	48.4 ± 0.6	0.535	49.3 ± 0.6	46.8 ± 0.5	0.004 *
Adequacy													
Total Fruits (5)	2.3 ± 0.1	2.2 ± 0.1	2.4 ± 0.1	0.47	2.3 ± 0.1	2.3 ± 0.1	0.851	2.2 ± 0.1	2.4 ± 0.1	0.12	2.5 ± 0.1	2.1 ± 0.1	0.004 *
Whole Fruits (5)	2.2 ± 0.1	2.0 ± 0.1	2.1 ± 0.1	0.252	2.2 ± 0.1	2.2 ± 0.1	0.739	2.0 ± 0.1	2.3 ± 0.1	0.094	2.4 ± 0.1	2.0 ± 0.1	0.012 *
Total Vegetables (5)	2.6 ± 0.1	2.6 ± 0.1	2.6 ± 0.1	0.893	2.6 ± 0.1	2.6 ± 0.1	0.813	2.7 ± 0.1	2.6 ± 0.1	0.519	2.7 ± 0.1	2.5 ± 0.1	0.153
Greens and Beans (5)	1.2 ± 0.1	1.4 ± 0.1	1.2 ± 0.1	0.464	1.4 ± 0.1	1.2 ± 0.1	0.227	1.3 ± 0.1	1.3 ± 0.1	0.54	1.3 ± 0.1	1.2 ± 0.1	0.356
Whole Grains (10)	2.4 ± 0.1	2.3 ± 0.2	2.5 ± 0.3	0.485	2.5 ± 0.1	2.3 ± 0.1	0.155	2.4 ± 0.1	2.4 ± 0.1	0.834	2.6 ± 0.1	2.2 ± 0.1	0.068
Dairy (10)	6.7 ± 0.1	6.7 ± 0.2	6.3 ± 0.2	0.444	6.5 ± 0.1	6.7 ± 0.2	0.293	6.3 ± 0.1	7.0 ± 0.2	0.057	6.7 ± 0.2	6.5 ± 0.1	0.342
Total protein foods (5)	4.0 ± 0.1	4.1 ± 0.1	4.2 ± 0.1	0.19	4.0 ± 0.1	4.0 ± 0.1	0.994	4.1 ± 0.1	3.9 ± 0.1	0.438	4.0 ± 0.1	4.0 ± 0.0	0.902
Seafood and Plant Proteins (5)	2.1 ± 0.1	1.8 ± 0.2	1.7 ± 0.1	0.049	2.0 ± 0.1	2.0 ± 0.1	0.42	2.0 ± 0.1	2.0 ± 0.1	0.79	2.1 ± 0.1	1.9 ± 0.1	0.343
Fatty Acids (10)	4.4 ± 0.1	4.3 ± 0.2	4.8 ± 0.2	0.282	4.5 ± 0.1	4.3 ± 0.1	0.502	4.6 ± 0.1	4.3 ± 0.2	0.768	4.4 ± 0.2	4.4 ± 0.1	0.603
Moderation													
Refined Grains (10)	4.7 ± 0.1	5.1 ± 0.3	5.2 ± 0.2	0.024	5.0 ± 0.1	4.8 ± 0.2	0.101	5.2 ± 0.1	4.6 ± 0.2	<0.001 *	5.1 ± 0.2	4.7 ± 0.1	0.169
Sodium (10)	4.2 ± 0.2	4.0 ± 0.2	3.8 ± 0.2	0.385	4.0 ± 0.1	4.3 ± 0.2	0.179	4.0 ± 0.1	4.2 ± 0.2	0.486	4.0 ± 0.2	4.2 ± 0.1	0.344
Added Sugars (10)	5.4 ± 0.1	5.5 ± 0.3	5.2 ± 0.2	0.449	5.4 ± 0.1	5.4 ± 0.1	0.623	5.1 ± 0.1	5.7 ± 0.1	0.002 *	5.5 ± 0.1	5.3 ± 0.2	0.221
Saturated Fats (10)	5.8 ± 0.1	5.7 ± 0.2	6.2 ± 0.2	0.129	6.0 ± 0.1	5.8 ± 0.1	0.473	6.1 ± 0.1	5.6 ± 0.1	0.172	6.1 ± 0.2	5.7 ± 0.1	0.051

Note: ^&^ *p*-values were obtained by performing PROC SURVEYREG, adjusted for age, race/ethnicity, parental education level, and the ratio of family income to poverty; ^a.^ normal weight vs. obese with *p* < 0.003846; PA, physical activity; HEI, the Healthy Eating Index; ^#^ Bonferroni correction was applied to all multiple comparisons between weight status with *p* < 0.003846 to be significant; ^$^ *p* < 0.003846; ^*^ *p* < 0.05.

**Table 4:** Initial analysis of interaction of weight related and physical activity variables and race/ethnicity for total HEI-2015 scores (*n* = 3373).

	Male (*n* = 1711)		Female (*n* = 1662)	
	β (95% CI)	*p*-Value	β (95% CI)	*p*-Value
**Model 1**				
Race/ethnicity		0.543		0.103
Hispanic	REF		REF	
non-Hispanic white	−1.13 (−7.85 to 5.60)		−4.43 (−10.46 to 1.60)	
non-Hispanic black	−3.17 (−9.02 to 2.68)		−5.75 (−11.32 to −0.19)	
BMI	0.04 (−0.13 to 0.21)	0.683	−0.06 (−0.25 to 0.14)	0.72
BMI * race/ethnicity		0.783		0.741
Hispanic	REF		REF	
non-Hispanic white	−0.07 (−0.33 to 0.19)		0.03 (−0.21 to 0.26)	
non-Hispanic black	0.01 (−0.23 to 0.25)		0.08 (−0.13 to 0.30)	
**Model 2**				
Race/ethnicity		0.026 ^#^		<0.001 ^#^
Hispanic	REF		REF	
non-Hispanic white	−2.70 (−4.90 to −0.50)		−3.72 (−6.09 to −1.35)	
non-Hispanic black	−2.30 (−4.22 to −0.38)		−4.05 (−6.07 to −2.03)	
Weight loss attempt	0.82 (−1.15 to 2.78)	0.728	0.35 (−1.77 to 2.46)	0.284
Weight loss attempt * race/ethnicity		0.488		0.83
Hispanic	REF		REF	
non-Hispanic white	−0.18 (−3.35 to 2.98)		−0.06 (−3.69 to 3.57)	
non-Hispanic black	−1.59 (−4.57 to 1.39)		0.80 (−2.32 to 3.92)	
**Model 3**				
Race/ethnicity		0.058		0.023 ^#^
Hispanic	REF		REF	
non-Hispanic white	−1.41 (−3.86 to 1.05)		−2.86 (−5.52 to −0.20)	
non-Hispanic black	−2.57 (−4.67 to −0.46)		−3.02 (−5.25 to −0.78)	
Weight dissatisfaction	1.13 (−0.79 to 3.04)	0.956	0.66 (−1.39 to 2.70)	0.734
Weight dissatisfaction * race/ethnicity		0.115		0.619
Hispanic	REF		REF	
non-Hispanic white	−2.89 (−5.79 to 0.01)		−1.67 (−5.29 to 1.94)	
non-Hispanic black	−0.58 (−3.40 to 2.24)		−1.01 (−4.19 to 2.17)	
**Model 4**				
Race/ethnicity		0.161		<0.001 ^#^
Hispanic	REF		REF	
non-Hispanic white	−1.81 (−4.62 to 1.01)		−6.43 (−8.83 to −4.04)	
non-Hispanic black	−1.82 (−4.03 to 0.39)		−4.51 (−6.21 to −2.80)	
Met PA recommendation	0.89 (−1.19 to 2.97)	0.711	−0.82 (−2.76 to 1.12)	0.655
Met PA recommendation * race/ethnicity		0.353		0.011 ^#^
Hispanic	REF		REF	
non-Hispanic white	−1.60 (−4.63 to 1.43)		5.09 (1.67 to 8.51)	
non-Hispanic black	−1.79 (−4.67 to 1.09)		2.16 (−0.70 to 5.01)	

Note: Parameter estimates (95% CI) and *p*-values were obtained by performing multiple PROC SURVEYREG, adjusted for age, sex, parental education level, and the ratio of family income to poverty; HEI, the Healthy Eating Index; BMI, body mass index; PA, physical activity; ^#^ *p* < 0.05.

**Table 5:** Final model of interaction of weight related and physical activity variables and race/ethnicity for total HEI-2015 scores (*n* = 3373).

	Male (*n* = 1711)		Female (*n* = 1662)	
	β (95% CI)	*p*-Value	β (95% CI)	*p*-Value
Race/ethnicity		<0.001 ^#^		<0.001 ^#^
Hispanic	REF		REF	
non-Hispanic white	−2.88 (−4.64 to −1.12)		−6.43 (−8.83 to −4.04)	
non-Hispanic black	−2.94 (−4.40 to −1.49)		−4.51 (−6.21 to −2.80)	
Met PA recommendation			−0.82 (−2.76 to 1.12)	0.401
Met PA recommendation * race/ethnicity				0.011 ^#^
Hispanic			REF	
non-Hispanic white			5.09 (1.67 to 8.51)	
non-Hispanic black			2.16 (−0.70 to 5.01)	

Note: Parameter estimates (95% CI) and *p*-values were obtained by performing multivariate PROC SURVEYREG. Race/ethnicity and significant interaction terms from Table 4 were put in the multivariate model, with age, sex, parental education level, and the ratio of family income to poverty included as covariates in all the models; HEI, the Healthy Eating Index; PA, physical activity; ^#^ *p* < 0.05.

**Table 6:** Determine the independent effects of race/ethnicity on total HEI-2015 scores (*n* = 3373).

	Total HEI-2015 Score		
	R^2^ (%)	ΔR^2^ (%)	Sig. ΔF
**Male (*n* = 1711)**			
Model 1: Demographic variables (age, parental education level, and family income)	0.5	0.5	0.0557
Model 2: Demographic variables, weight-related variables, and PA	0.9	0.4	0.3131
Model 3: Demographic variables, weight-related variables, PA, and race/ethnicity	1.7	0.8	0.0004 *
**Female (*n* = 1662)**			
Model 1: Demographic variables (age, parental education level, and family income)	0.9	0.9	0.0031
Model 2: Demographic variables, weight-related variables, and PA	2.3	1.4	0.0014 *
Model 3: Demographic variables, weight-related variables, PA, and race/ethnicity	3.6	1.3	<0.0001 *

Note: R-squared was obtained by performing multiple PROC REG with weight statement which is the percentage of the response variable variation that is explained by a linear model. The predictor variables that is associated with the greatest increase in R-squared are important variables. We used sequence models to look at the change in R-squared; Model 1: included age, sex, parental education level and PIR; Model 2: added weight-related variables and physical activity into model 1; Model 3: added race/ethnicity into model 2; HEI = the Healthy Eating Index; ^#^ *p* < 0.05.

**Table 7:** Determine the independent effects of weight-related variables and physical activity on total HEI-2015 scores stratifying by race/ethnicity (*n* = 3373).

	Non-Hispanic Whites			Non-Hispanic Blacks			Hispanics		
	R^2^ (%)	ΔR^2^ (%)	Sig. ΔF	R^2^ (%)	ΔR^2^ (%)	Sig. ΔF	R^2^ (%)	ΔR^2^ (%)	Sig. ΔF
**Male (*n* = 1711)**									
Modle1: Age, sex, parental education level and PIR	0.8	0.8	0.2223	1.6	1.6	0.0707	0.2	0.2	0.7252
Model 2: Age, sex, parental education level, PIR, and BMI	0.8	0.0	0.7884	1.9	0.3	0.2720	0.2	0.0	0.8519
Model 3: Age, sex, parental education level, PIR, BMI, and weight loss attempt	0.9	0.1	0.4938	2.4	0.5	0.1122	0.6	0.4	0.1804
Model 4: Age, sex, parental education level, PIR, BMI, weight loss attempt, and weight dissatisfaction	1.7	0.8	0.0456 *	2.5	0.1	0.5166	0.6	0.0	0.5123
Model 5: Age, sex, parental education level, PIR, BMI, weight loss attempt, Weight dissatisfaction, and met PA recommendation	1.7	0.0	0.5788	2.7	0.2	0.4660	0.7	0.1	0.5967
**Female (*n* = 1662)**									
Modle1: Age, sex, parental education level and PIR	3.6	3.6	0.0008 *	0.7	0.7	0.3978	0.4	0.4	0.5388
Model 2: Age, sex, parental education level, PIR, and BMI	3.6	0.0	0.7987	0.7	0.0	0.7481	0.5	0.1	0.3729
Model 3: Age, sex, parental education level, PIR, BMI, and weight loss attempt	3.6	0.0	0.5671	0.9	0.2	0.3845	0.6	0.1	0.4656
Model 4: Age, sex, parental education level, PIR, BMI, weight loss attempt, and weight dissatisfaction	3.9	0.3	0.2668	1.1	0.2	0.3460	0.8	0.2	0.3278
Model 5: Age, sex, parental education level, PIR, BMI, weight loss attempt, Weight dissatisfaction, and met PA recommendation	6.5	2.6	0.0004 *	1.7	0.6	0.0998	0.8	0.0	0.6066

Note: R-squared was obtained by performing multiple PROC REG with weight statement, which is the percentage of the response variable variation that is explained by a linear model. The predictor variables associated with the greatest increase in R-squared are important variables. We used sequence models to look at the change in R-squared; Model 1 included age, sex, parental education level, and PIR; Model 2 added BMI into Model 1; Model 3 added weight loss attempt into Model 2; Model 4 added weight dissatisfaction into Model 3; Model 5 added met physical activity recommendation into Model 4; HEI, the Healthy Eating Index; PA, physical activity; PIR, the ratio of family income to poverty; BMI, body mass index; * *p* < 0.05.

## Supplementary Materials

The following are available online at https://www.mdpi.com/1660-4601/16/23/4803/s1. Table S1: Respondents characteristics by sex.

## Declaration

All data are publicly accessible and available in the NHANES repository, https://wwwn.cdc.gov/nchs/nhanes/sasviewer.aspx.
