# Supplementary material for: Racial/Ethnic Disparities in US Adolescents’ Dietary Quality and Its Modification by Weight-Related Factors and Physical Activity
Source: Int J Environ Res Public Health. 2019 Nov 29;16(23):4803. doi: 10.3390/ijerph16234803 (PMC6926511; doi:10.3390/ijerph16234803)
Supplement: Supplementary file 1 [file ijerph-16-04803-s001.pdf]

Supplementary Table S1. Respondents characteristics by sex (n = 3373).

	Male	Female	*p*-Value
	**n = 1711(50.5%)**	**n = 1662(49.5%)**	
Age (yrs)	15.4 ± 0.1	15.4 ± 0.1	0.933
Race/ethnicity			
Hispanic	576 (63.5)	506 (62.4)	0.476
Non-Hispanic White	483 (15.0)	485 (15.8)	0.420
Non-Hispanic Black	652 (21.6)	671 (21.8)	0.843
Parent education level, n (weighted %)			
High school or less	850 (39.9)	856 (42.9)	0.158
College or above	800 (60.1)	754 (57.1)	0.158
PIR, n (weighted %)			
<1.0	482 (20.3)	528 (23.4)	0.108
≥ 1.0	1112 (79.7)	995 (76.6)	0.108
Body mass index (kg/m2)	24.1 ± 0.2	24.3 ± 0.2	0.455
Actual weight status, n (weighted %)			
Normal weight	1021 (61.1)	954 (63.1)	0.648
Overweight	290 (17.0)	323 (17.0)	0.875
Obese	400 (21.8)	385 (20.0)	0.378
Weight loss attempt, n (weighted%)	647 (35.6)	857 (47.8)	<0.001*
Weight dissatisfied, n (weighted %)	926 (50.7)	946 (52.0)	0.259
Met PA recommendation, n (weighted %)	1112 (68.9)	746 (50.9)	<0.001*
HEI-2015 (100)	46.3±0.4	48.0±0.3	0.001*
Adequacy			
Total Fruits (5)	2.1 ± 0.1	2.3 ± 0.1	0.006*
Whole Fruits (5)	1.9 ± 0.1	2.2 ± 0.1	0.007*
Total Vegetables (5)	2.2 ± 0.0	2.6 ± 0.0	<0.001*
Greens and Beans (5)	1.0 ± 0.1	1.3 ± 0.1	<0.001*
Whole Grains (10)	2.3 ± 0.1	2.4 ± 0.1	0.481
Dairy (10)	7.0 ± 0.1	6.6 ± 0.1	0.002*
Total protein foods (5)	4.2 ± 0.0	4.0 ± 0.0	<0.001*
Seafood and Plant Proteins (5)	1.8 ± 0.1	2.0 ± 0.1	0.043*
Fatty Acids (10)	3.8 ± 0.1	4.4 ± 0.1	<0.001*
Moderation			
Refined Grains (10)	4.8 ± 0.1	4.9 ± 0.1	0.916
Sodium (10)	4.1 ± 0.1	4.1 ± 0.1	0.791
Added Sugars (10)	5.3 ± 0.1	5.4 ± 0.1	0.308
Saturated Fats (10)	5.7 ± 0.1	5.9 ± 0.1	0.072

Note: Data are present as weighted Mean± standard error for continuous variables and frequencies and weighted proportions for categorical variables; *p*-value for continuous variables was obtained by performing PROC SURVEYREG, and *p*-value for category variable was obtained by performing PROC SURVEYLOGISTC; except for sex, age, parental education level and family income comparisons which were not performed adjusted analysis, all the other variable comparisons were adjusted for age, race/ethnicity, parental education level and PIR; PA, physical activity; PIR, the ratio of family income to poverty; HEI, the Healthy Eating Index; $^{*}p < 0.05$.
